# Accelerating Medicines Partnership® Parkinson's Disease Proteomics: A Comprehensive Resource for Advancing Parkinson's Disease Research

**DOI:** 10.1002/mds.70183

**Published:** 2026-02-05

**Authors:** Victoria J. Dardov, Aparna Vasanthakumar, Ameya Kulkarni, Niveda Sundararaman, Rakhi Pandey, Preeti Bais, Jennifer E. Van, Maria Quinton, Barry Landin, David Vismer, Bradford Casey, Matt Bookman, Willy Nojopranoto, Erin Teeple, Can Kayatekin, Rajaraman Krishnan, Richard Hargreaves, Guhan Nagappan, S. Pablo Sardi, Sri Ramulu Pullagura, Christine Swanson‐Fischer, Accelerating Medicines Partnership Parkinson's Disease (AMP PD) Proteomics Working Group

**Affiliations:** ^1^ Technome Herndon Virginia USA; ^2^ AbbVie North Chicago Illinois USA; ^3^ Smidt Heart Institute, Cedars‐Sinai Medical Center Los Angeles California USA; ^4^ Sanofi Cambridge Massachusetts USA; ^5^ The Michael J. Fox Foundation for Parkinson's Research New York New York USA; ^6^ Verily Life Sciences San Jose California USA; ^7^ Neuroscience Thematic Research Center, Bristol Myers Squibb Cambridge Massachusetts USA; ^8^ Glaxo Smith Kline Collegeville Pennsylvania USA; ^9^ Foundation for the National Institutes of Health North Bethesda Maryland USA; ^10^ National Institute of Neurological Disorders and Stroke Bethesda Maryland USA

## Abstract

**Background:**

Recent advances in proteomic profiling have enabled its use as a powerful approach in elucidating molecular mechanisms underlying Parkinson's disease, enabling the identification of disease‐associated protein alterations and candidate biomarkers for diagnosis, progression, and therapeutic response.

**Objectives:**

The Accelerating Medicines Partnership® Parkinson's Disease (AMP PD) program is a public–private partnership between the National Institutes of Health (NIH), multiple biopharmaceutical and life sciences companies, and non‐profit organizations, managed by the Foundation for National Institutes of Health (FNIH). The program aims to advance the molecular and clinical characterization of PD through deep, longitudinal profiling of patient data and biosamples, with the goal of identifying and validating diagnostic, prognostic, and progression biomarkers for PD.

**Methods:**

Longitudinal proteomic profiling, both targeted and untargeted, was performed on cerebrospinal fluid (CSF) and plasma samples. The resulting datasets are publicly accessible via the AMP® PD Knowledge Platform.

**Results:**

The proteomic datasets enable differential protein analyses and can be used to explore molecular alterations associated with PD progression and heterogeneity.

**Conclusions:**

These studies contribute to the broader AMP PD initiative by providing the research community with a harmonized proteomics dataset. As part of the larger AMP PD data, this work provides the PD community with a harmonized and accessible proteomics dataset that can be utilized for discovery, hypothesis generation, and validation of users’ own research. © 2026 Technome. *Movement Disorders* published by Wiley Periodicals LLC on behalf of International Parkinson and Movement Disorder Society. This article has been contributed to by U.S. Government employees and their work is in the public domain in the USA.

Proteomics is the large‐scale study of proteins and gives an understanding of the dynamism of proteins.^1^ With technological development in the proteomics field,^2^ large‐scale proteomic profiling of biofluids for biomarker discovery is now a possibility, specifically when it comes to expression proteomics.^1^ Advancements in both mass spectrometers and affinity‐based platforms are driving proteome coverage and throughput, allowing for quantification of more proteins in more biological samples,^3^ and additionally, it is now feasible to conduct single‐cell proteomics studies utilizing highly‐sensitive instruments.^4^ As the field continues to evolve rapidly, applying these cutting‐edge approaches to the study of neurodegeneration represents a logical and promising next step. Such applications hold significant potential to elucidate the molecular and cellular underpinnings of these complex diseases, ultimately informing both biomarker discovery and therapeutic development.

## Parkinson's Disease

Parkinson's disease (PD) is a heterogenous disorder characterized by a wide spectrum of etiologies, pathogenic contributors, and clinical manifestations. Approximately 3–5% PD cases are associated with monogenic causes linked to known PD genes. In contrast, most PD cases are idiopathic and influenced by complex genetic architectures. Recent genome‐wide association studies have identified over 90 other genetic risk variants accounting for 16–36% of the heritable risk of non‐monogenic PD.^5^

The heritable risk for PD has been associated with several genes, including *SNCA, LRRK2, Parkin, PINK1, DJ‐1, ATP13A2, MAPT, GBA1*, and others involved in diverse biological pathways implicated in PD pathogenesis and neurodegeneration more broadly.^6^, ^7^ Despite the absence of genetic risk factors in many cases, patients with idiopathic PD have been shown to harbor proteomic phenotypes such as those observed in monogenic forms of the disease.^8^ Similarly, elevated LRRK2 kinase activity, a hallmark of PD‐associated *LRRK2* mutations, has also been observed in PD patients without such mutations.^9^ These findings suggest that complex polygenic influences may converge on common proteomic alterations, thereby recapitulating aspects of monogenic PD at the molecular level.

Thus, complex multigenic traits may lead to changes to the proteome similar to those arising from monogenic risk factors associated with PD. Measuring proteomic changes at a large scale in well‐characterized cohorts may provide deeper insights into the underlying causes of PD, identify novel biomarkers, and support targeted therapeutic approaches.

## Proteomics in PD Research

Previous work on proteomics profiling in PD has identified several promising candidate biomarkers for diagnosis, by examining differential proteomic expression levels in postmortem brain tissue, cerebrospinal fluid (CSF), and peripheral blood from PD cases and controls.^7^, ^10^, ^11^, ^12^, ^13^, ^14^, ^15^ Many of these differentially expressed proteins are associated with biological pathways implicated in PD pathogenesis, including oxidative stress, mitochondrial dysfunction, impaired protein degradation, and inflammation.^7^, ^10^, ^11^, ^12^, ^13^, ^14^, ^15^ Despite these advances, there remains a critical need to validate these findings in larger independent cohorts and establish robust associations between protein expression profiles and clinically relevant outcomes. Such efforts are essential for translating proteomic discoveries into actionable diagnostic and therapeutic tools.

## Accelerating Medicines Partnership® Parkinson's Disease

Robust validation using large independent datasets is particularly critical in PD, a disease characterized by considerable heterogeneity in both genetic etiology and clinical progression. The proteomics dataset generated by the Accelerating Medicines Partnership® Parkinson's Disease (AMP PD) initiative represents an unprecedented resource for both discovery and validation in PD proteomic research. This resource enables high‐resolution analyses of temporal changes in protein expression in peripheral blood and CSF, assessment of inter‐individual variability in expression profiles, and integration of proteomic signatures with harmonized clinical and multi‐omic data.

A hallmark of AMP PD is its commitment to open science and data sharing. This includes the dissemination of comprehensive PD‐associated datasets hosted in the AMP® PD Knowledge Platform, such as proteomics data from both the Parkinson's Progression Markers Initiative (PPMI) and the Parkinson's Disease Biomarkers Project (PDBP) cohorts. A core objective is to develop datasets that are of broad utility to the research community. Consequently, both raw and aggregated data are made available to accommodate users across a spectrum of expertise, from novice to advanced computational scientists.

At the time of writing, the AMP PD Knowledge Platform includes harmonized clinical data for all participants, as well as whole genome sequencing (WGS) data for 10,590 joint genotyped samples, bulk transcriptomics data for 4123 participants comprising 10,609 whole blood bulk RNA samples, proteomic profiles from 695 individuals captured through targeted and untargeted assays, and postmortem single‐nucleus RNAseq data from 100 participants spanning five brain regions and linked to WGS and clinical metadata.

Building on the success of the first phase, AMP PD has recently launched the Accelerating Medicines Partnership® in Parkinson's Disease and Related Disorders (AMP® PDRD). This expanded effort aims to incorporate related synucleinopathies and atypical parkinsonian disorders such as dementia with Lewy bodies (DLB), multiple system atrophy (MSA), rapid eye movement (REM) behavioral disorder (RBD), and progressive supranuclear palsy (PSP). The goal of AMP PDRD is to facilitate biomarker discovery and validation, advance multi‐omic molecular profiling, and refine endophenotyping strategies across a broader neurodegenerative spectrum.

## Complementary Programs

In addition to AMP PD, several joint programs and collaborative consortia have significantly advanced our understanding of PD and broader neurodegenerative biology through data‐centric approaches.

The Global Neurodegeneration Proteomics Consortium (GNPC) is an ambitious, collaborative neurodegenerative disease biomarker discovery initiative, uniting academic, governmental, and industry partners. The GNPC's Version 1 Harmonized Data Set (HDS) comprises proteomic data from more than 35,000 patient samples contributed by over 20 international cohorts, totaling nearly 250 million unique protein measurements.^16^ Covering Alzheimer's disease (AD), PD, amyotrophic lateral sclerosis, and frontotemporal dementia, the HDS enables both longitudinal and cross‐sectional analyses and is the largest proteomic‐based biomarker dataset available for neurodegeneration. Through integrated proteomic and proteogenomic analyses, the GNPC aims to elucidate both disease‐specific and shared molecular mechanisms.

Complementing these efforts is the UK Biobank Pharma Proteomics Project, a landmark initiative focused on mapping the plasma proteome at unprecedented scale. By measuring approximately 1500 traditionally elusive proteins in nearly 53,000 participants, the project is generating a foundational resource to explore associations between protein expression and a wide range of clinical phenotypes.^17^

Within this evolving landscape, AMP PD contributes a unique and richly annotated dataset that supports the growing demand for large‐scale, harmonized, and clinically integrated proteomics resources in PD research. This resource article provides an overview of the proteomics data available through AMP PD, offers guidance for data access and analysis, and highlights case studies and tools developed to facilitate the use of these data by the broader research community.

## Patients and Methods

### Cohorts

AMP PD Release 4 utilized plasma and CSF samples from participants enrolled in PDBP (https://pdbp.ninds.nih.gov/) and PPMI (https://www.ppmi-info.org/). All participants provided written informed consent, and all protocols received local institutional review board and ethical approvals. Further cohort‐specific details are available via the AMP PD website (https://amp-pd.org) and respective cohort study webpages.

### Sample Collection

Sample collection and biobanking are supported through BioSEND and MJFF Biorepository with shared infrastructure and harmonized protocols at Indiana University. Detailed biobanking protocols can be accessed for PDBP and PPMI.

### Sample Selection Criteria

CSF samples with a hemoglobin contamination <100 ng were prioritized for untargeted proteomics analysis to minimize interference from blood proteins. To enable longitudinal analysis, subjects were selected based on the availability of matched plasma and CSF at a minimum of three timepoints, as well as corresponding clinical and multi‐omic data in AMP PD.

Although the Olink® platform did not require low hemoglobin levels in CSF, matched subjects and samples (where possible) were included in both targeted and untargeted analyses to facilitate direct comparisons. Participant samples from PPMI and PDBP cohorts included PD and healthy controls, which were prioritized based on availability of multiple timepoints for longitudinal analysis. While not specifically selected, there are a small number of prodromal participants and participants with known PD genetic mutations within the proteomics dataset. Participants were not excluded based on medication usage, and therefore potential medication effects on protein levels should be considered in downstream statistical analyses.

### Targeted Proteomics Methods

See supplementary materials in Appendix S1 for detailed methods. Standard Olink® Explore 1536 (Olink Proteomics AB, Uppsala, Sweden) assay was performed.

### Untargeted Proteomics Methods

See supplementary materials in Appendix S1 for detailed methods. Standard operating procedures (SOPs) were utilized for sample processing, data‐independent acquisition (DIA), and data processing.

### AMP PD Data Curation

Data received from providers were reformatted into standard AMP PD structure, incorporating AMP PD participant identifiers and proteomics sample IDs. All proteomics data were harmonized to align with existing AMP PD infrastructure and naming conventions.

### Data Availability

Proteomics data processing and quality control were performed by Olink, AbbVie, and the Jennifer Van Eyk laboratory at Cedars‐Sinai Medical Center, which were also responsible for data curation and reformatting into AMP PD‐compatible formats. All processed data including normalized protein expression (NPX) files for targeted proteomics, and raw (.raw, .wiff), mzML (.mzML), and fragment‐, peptide‐, and protein‐level files for untargeted proteomics are hosted on the Google Cloud Platform.

### Resource Availability

All AMP PD proteomics resources are available on the Terra platform and require Tier 2 AMP PD data access. Approved users can view, access, and clone available workspaces and analytical notebooks for their own use.

### Additional Methods

Detailed protocols for targeted and untargeted proteomics are available on the AMP PD website (targeted proteomics: https://amp-pd.org/data/targeted-proteomics-data; untargeted proteomics: https://amp-pd.org/data/untargeted-proteomics-data).

### Analysis Overview

To support data reuse, example analyses using both targeted and untargeted proteomics datasets have been made available. These analyses illustrate practical applications of the AMP PD proteomics data.

### Proteomics Participant Demographics

An overview of AMP PD proteomics participants and samples in provided in Table 1, summarizing demographic characteristics such as average age at baseline, sex, race, and clinical condition. The proteomics data are divided into two categories: Proteomics Data‐Independent Acquisition (PDIA) and Proteomics Proximity Extension Assay (PPEA). PDIA refers to mass spectrometry‐based data acquired through data‐independent acquisition and includes both CSF and plasma samples. PPEA refers to Olink‐generated data using proximity extension assay technology and is available for both CSF and plasma; the current dataset is designated ‘D03,’ indicating the third PPEA dataset released by AMP PD. Due to longitudinal sampling, the total number of samples exceeds the number of unique participants.

**TABLE 1:** Participant demographics (including average and standard deviation for age at baseline) in Proteomics Data‐Independent Acquisition (PDIA) mass spectrometry‐derived data and Proteomics Proximity Extension Assay Olink® Platform Dataset 03 (PPEA‐D03) data.

Parameter	CSF‐PDIA n	PLA‐PDIA n	CSF‐PPEA‐D03 n	PLA‐PPEA‐D03 n
Total samples	2761	1451	1297	1529
Total participants	621	317	398	413
Age at baseline (years)				
Average	61.87	62.24	63.39	63.98
SD	9.58	9.61	9.63	9.47
Sex				
Female	230	114	140	142
Male	391	203	258	271
Race				
Asian	7	1	3	3
Black or African American	11	6	9	10
Multiracial	9	5	6	6
Other	4	3	3	3
Unknown	2		5	5
White	588	302	372	386
Condition				
Case	365	189	175	181
Control	209	112	178	179
Other	47	16	45	53

*Note*: Data are available for both CSF and plasma.

Abbreviations: CSF, cerebrospinal fluid; PLA, plasma; SD, standard deviation.

For each participant in the proteomics data, matched plasma and CSF across longitudinal timepoints were utilized (Supplementary Table S1). The distribution of visit months is centered around the primary scheduled intervals of 0, 12, 24, 36, 48, and 60 months, with additional intermediate and extended timepoints represented. This temporal structure is consistent across both the PDIA and PPEA proteomics platforms and applies to CSF and plasma samples. In addition, there is overlap between samples and participants between PDIA and PPEA and CSF and plasma (Supplementary Fig. S1), showing that some longitudinal data do overlap between the different tissue types and data types.

WGS data are available for each participant within the proteomics experiments and can be used to determine if participants had a known mutation such as *SNCA* (A53T [rs104893877]), *LRRK2* (G2019S [rs34637584]; R1441C_T [rs33939927]; R1441G_G [rs33939927]) *or GBA1 (*N370S [rs76763715]; T369M [rs75548401]; E326K [rs2230288]) and which variant of APOE was present. Supplementary Table S2 shows the number of samples that do or do not have a PD associated mutation. For APOE, those marked ‘Yes’ for having an APOE variant are either homozygous or heterozygous for the E4 variant, which has been demonstrated to increase the risk of AD and AD‐related dementias.

### AMP PD Proteomics Analysis Use Case 1 (Targeted)

A total of 2872 samples were analyzed using the targeted Olink Platform, which included 1320 samples from CSF and 1552 samples from plasma at 16 visits as shown in Supplementary Table S1. Case–control differential expression analysis was performed separately for CSF and plasma samples.

**FIG. 1:**
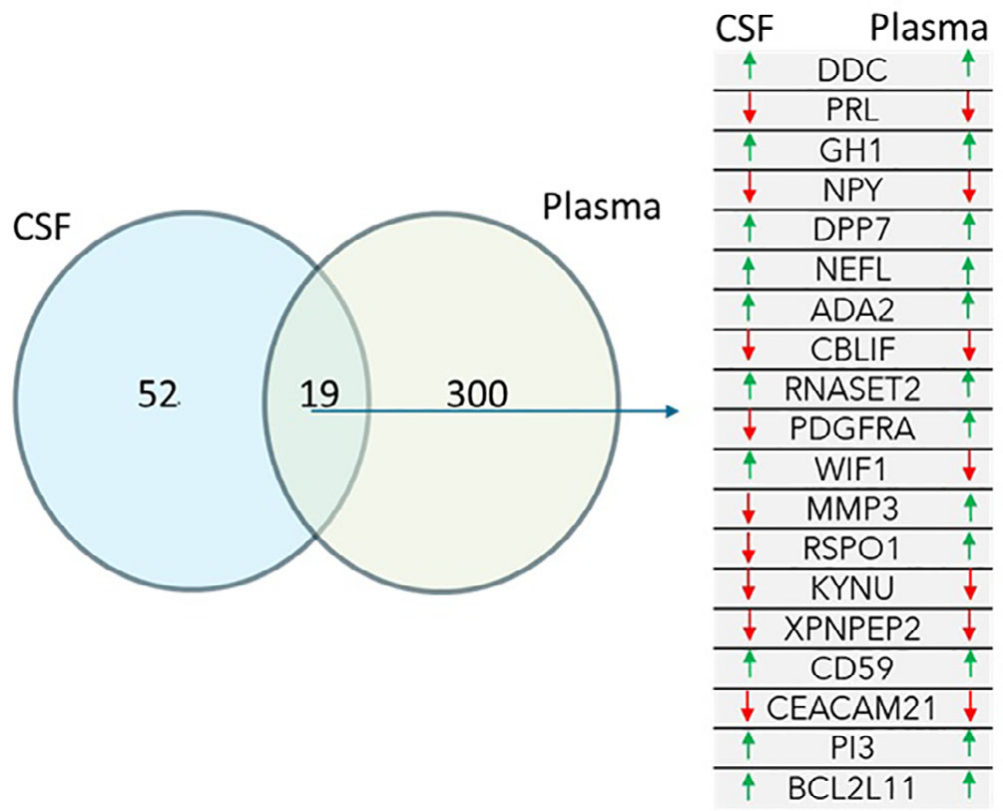
Differential protein expression analysis revealed partial overlap between cerebrospinal fluid (CSF) and plasma, with a subset of proteins found to be significantly differentially expressed in both matrices.

A Welch's two‐sample *t*‐test was conducted for each protein, comparing expression levels between samples labeled as Case and Control. This analysis identified 71 significantly differentially expressed proteins in CSF and 319 proteins in plasma at a nominal *P*‐value threshold of ≤0.05. Of these, there was an overlap of 19 proteins that were significantly differentially expressed in both plasma and CSF as shown in Figure 1.

The next set of analyses examined differences between the two cohorts represented in the targeted proteomics datasets (PPMI and PDBP) to assess the consistency of differentially expressed proteins across these cohorts.

Among the 1320 CSF samples, 521 were from the PDBP cohort and 799 from PPMI. Case–control differential expression analysis using Welch's two‐sample *t*‐test identified 24 significantly differentially expressed proteins in the PDBP cohort and 25 proteins within the PPMI cohort (*P* ≤ 0.05). Seven proteins were found to be significantly differentially expressed in both cohorts in CSF as shown in Supplementary Figure S2.

These results indicate that cohort‐specific analyses yield fewer overlapping differentially expressed proteins compared with analyses performed on the combined dataset.

The 1552 samples within plasma consisted of 521 samples from the PDBP cohort and 1031 samples from PPMI cohort. Case–control differential expression analysis was conducted separately for each cohort using Welch's two‐sample *t*‐test, applied to each protein (by OlinkID). This analysis identified 161 significantly differentially expressed proteins in the PDBP cohort and 219 in the PPMI cohort (*P* ≤ 0.05). Notably, 32 proteins were significantly differentially expressed in both cohorts, as illustrated in Supplementary Figure S3.

These analyses indicate that plasma exhibits a greater number of differentially expressed proteins than CSF, both in the overall case–control comparison and in cohort‐specific analyses, suggesting higher sensitivity or broader signal detection plasma‐based proteomics.

### AMP PD Proteomics Analysis Use Case 2 (Untargeted)

Untargeted proteomics data from plasma and CSF were analyzed to identify differentially expressed proteins (DEPs) between clinical and genetic subgroups.

**FIG. 2:**
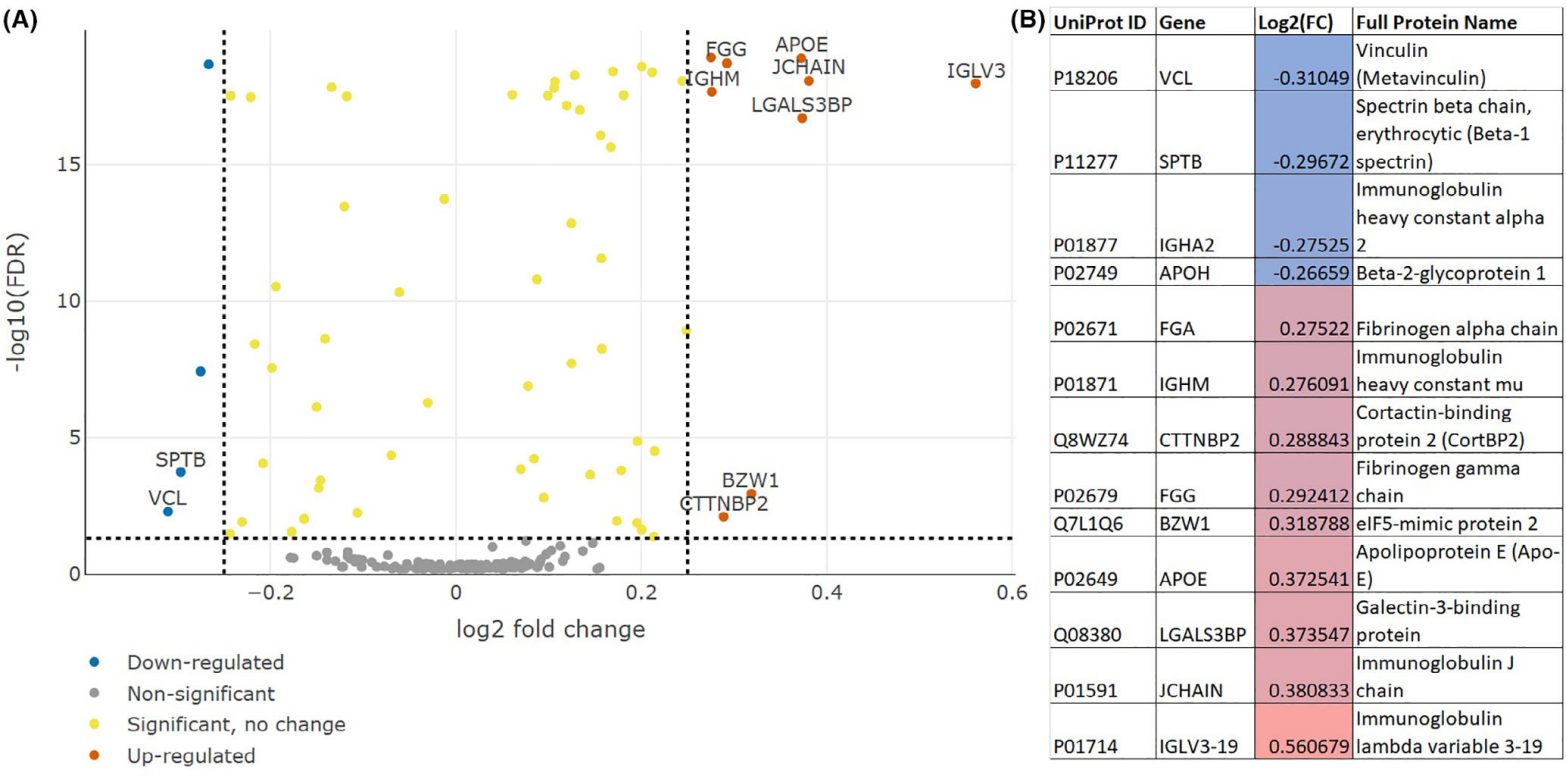
Analysis of plasma data comparing Parkinson's disease (PD) to other neurological disorders as classified by Accelerating Medicines Partnership® Parkinson's Disease (AMP PD) clinical data. (A) Volcano plot of the analysis shows upregulated and downregulated proteins. (B) Differentially expressed proteins are listed along with fold change and full protein name with proteins that are more abundant in PD compared with other neurodegenerative diseases colored in red and proteins that are less abundant in PD compared with other neurodegenerative diseases in blue.

The first comparison focused on patients diagnosed with *Other neurological diseases* versus *Parkinson's disease (PD) only*, using mapDIA on a filtered, batch‐corrected, and imputed dataset at the protein level (Fig. 2). A total of 13 DEPs met the predefined thresholds of |log₂ fold change| ≥0.25 and *P*‐value ≤0.05. Of these, four proteins were downregulated and nine were upregulated in the *Other* condition relative to *PD only*. Notably, downregulated proteins included muscle‐specific proteins (eg, *VCL*), while upregulated proteins were primarily derived from B cells (eg, immunoglobulins) and fibroblasts (eg, *FGA, FGG*).

A second analysis compared patients with and without genetic variants associated with PD (*GBA1*, *LRRK2*, *SNCA*, or *APOE ε4*). This comparison was performed at three timepoints (month 0, 24, and 48), selected based on sample availability and consistent spacing between visits (Supplementary Tables S3 and S4). Principal component analysis (PCA) of these groups revealed overlapping distributions along PC1 and PC2 across all timepoints (Supplementary Fig. S4A–C), suggesting that global proteomic profiles are broadly similar between mutation carriers and non‐carriers.

At the protein level, differential expression analysis identified two DEPs at month 0, none at month 24, and four DEPs at month 48, using the same significance and fold‐change criteria (Supplementary Fig. S5A–B). Interestingly, a consistent trend was observed: at month 0, *MST1* (a pro‐apoptotic protein) was downregulated in mutation carriers, while at month 48, *CD5L* (an inhibitor of apoptosis) was upregulated in the same group, suggesting potential alterations in apoptotic signaling. Despite these differences, PCA at each timepoint demonstrated no distinct clustering between mutation‐positive and mutation‐negative participants (Supplementary Fig. S4A–C).

In CSF, a parallel comparison of *Other neurological diseases* versus *PD only* also revealed distinct protein expression patterns (Supplementary Fig. S6). DEPs in CSF were enriched for apolipoproteins and immune response‐related proteins, with higher expression in the *Other* group. However, no overlap was observed between DEPs identified in CSF and those in plasma, indicating tissue‐specific proteomic signatures in this comparison.

### AMP PD Proteomics Analysis Use Case 3 (Baseline Targeted Proteomic Profiles in PD Mutation Carriers vs. Non‐Carriers)

**FIG. 3:**
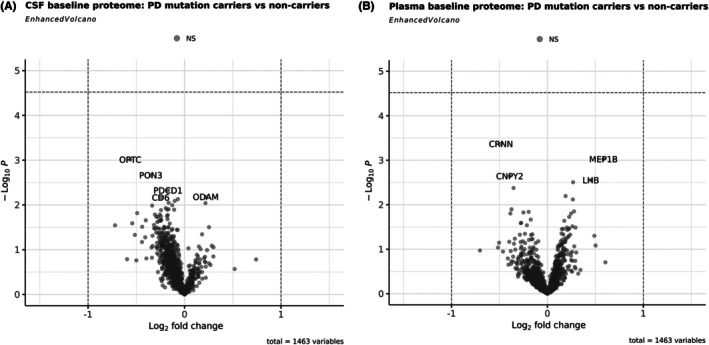
Volcano plots of plasma and cerebrospinal fluid (CSF) Proteomics Proximity Extension Assay Olink® Platform Dataset 03 (PPEA‐D03) data analysis. (A) Volcano plot of CSF Parkinson's disease (PD) mutation carriers versus non‐mutation carriers at baseline shows no significant differentially expressed proteins. (B) Volcano plot of plasma PD mutation carriers versus non‐mutation carriers at baseline shows no significant differentially expressed proteins.

Targeted proteomics data were used to compare baseline (month 0) protein expression between PD mutation carriers (*APOE ε4*, *GBA1*, and *LRRK2)*; see Supplementary Table S2) and non‐carriers in both CSF and plasma. The analysis revealed no statistically significant differences in protein expression between the groups. As shown in the volcano plots (Fig. 3A,B), no proteins met the predefined thresholds for both log₂ fold change and *P*‐value, indicating no detectable differential expression at baseline in this targeted dataset.

### AMP® PD Terra Resources: Analysis

To complement analyses presented within this article, an analysis workspace has been made available for Tier 2 AMP PD users, providing access to AMP PD data and R‐based Terra notebooks that allow users to reproduce the figures and results from this publication. The analysis workspace can be access through the AMP PD website.

### AMP® PD Terra Resources

To support user engagement with the available proteomics data, AMP PD has developed two dedicated Terra workspaces, accessible to users with Tier 2 access.

The ‘Getting Started Tier 2 – Proteomics’ workspace includes complementary R and Python notebooks tailored to the latest AMP PD data release (Supplementary Table S5). These notebooks are designed to help users locate and access proteomics data via Google cloud storage (GCS) or BigQuery, and to demonstrate how to integrate proteomics data with associated demographic and clinical variables.

The Proteomics Quality Control and Analysis workspace provides R notebooks (Supplementary Table S6) designed to familiarize users with fundamental analyses of both targeted and untargeted proteomics datasets.

Additional AMP PD and Terra resources can be found on the AMP® PD Featured Terra Workspace (https://app.terra.bio/#workspaces/amp-pd-public/AMP-PD-In-Terra).

### AMP PD Community

In addition to the AMP PD‐provided workspaces, community‐provided workspaces are available through the Terra Platform, featuring community provided analyses of AMP PD data (Supplementary Fig. S7).

## Discussion

In this article, we provide a comprehensive overview of the proteomics datasets available through Release 4.0 of the AMP PD program. The data include both targeted and untargeted proteomics, with extensive quality control, curation, and formatting to ensure usability for a broad range of research applications. Raw and processed data, including.mzML and raw files for mass spectrometry, are available to enable in‐depth analysis. To support accessibility, AMP PD interactive Terra Workspaces have preconfigured Jupyter Notebooks to streamline data exploration, visualization, and analysis in the cloud environment.

To demonstrate the utility of the dataset, we provide example use cases highlighting potential research questions that can be explored using AMP PD proteomics data. These examples illustrate that biologically meaningful insights can be derived from both targeted and untargeted platforms. Researchers can leverage these datasets to conduct longitudinal analysis, investigating protein and peptide level changes over time. Furthermore, the availability of harmonized clinical, genetic, and transcriptomic data enables the integration of proteomics with other data types, facilitating multi‐omic analyses and the identification of correlations with clinical phenotypes or genetic variants. This integrated resource can also support the discovery and validation of biomarker signatures associated with disease progression or response to therapy.

While AMP PD represents one of the most comprehensive proteomics resources for PD, additional efforts across the scientific community have also advanced proteomic biomarker discovery. Despite significant research, relatively few PD biomarkers have translated into clinical practice.^18^ Compared with AD, the field of PD has lagged in the availability of high‐throughput and large‐scale proteomics datasets. Recent initiatives, however, have begun to address this gap. Mass spectrometry‐based studies in independent cohorts of over 200 individuals have quantified approximately 1400 proteins in CSF and identified proteins dysregulated in PD, implicating inflammatory and lysosomal pathways. Within the PPMI, targeted proteomic profiling using the aptamer‐based platform SomaScan® in CSF samples from over 1100 individuals identified mitochondrial dysfunction and neuroinflammation as prominent features of idiopathic PD.^14^ Integrating proteomic data with genetic analyses (eg, protein quantitative trait loci, pQTLs) enabled the identification of potentially causal, druggable targets. These findings informed the development of a PD Proteomic Score (PD‐ProS), validated across independent cohorts.^19^ The Olink® Proximity Extension Assay, applied to plasma from PD patients, demonstrated immune dysregulation associated with disease severity.^20^ Additionally, in prodromal PD patients from the PPMI cohort, it revealed molecular signatures associated with endothelial dysfunction, thromboembolic processes, and neuroinflammation.^21^ Both studies utilized the Target 96 Olink® Panel, which measures 96 proteins per run. The AMP PD dataset includes matched longitudinal CSF and plasma samples from PD and healthy controls, enabling a deeper temporal understanding of protein changes across disease stages.

Despite the strengths of this AMP PD proteomics dataset, certain limitations must be acknowledged. Currently, the dataset is comprised primarily of participants from European ancestry, which may limit generalizability. With the launch of the second phase of the initiative, AMP PDRD, we anticipate additional heterogenous participants, enabling broader insights into proteomic variability and disease mechanisms across populations.

The targeted nature of Olink Panels impose constraints on discovery‐driven analyses, given the limited analyte. However, the high specificity and optimization for serum/plasma lend themselves to robust, relative quantification. Enabling technologies, such as the Nucleic acid Linked Immuno‐Sandwich (NULISA™), offer enhanced sensitivity and may expand future biomarker discovery efforts in detecting low abundant proteins in this nascent platform.^22^

Conversely, untargeted mass spectrometry provides broader analyte coverage but has lower sensitivity for detecting low‐abundance proteins. Moreover, these assays generate relative, rather than absolute, quantification, potentially limiting some types of downstream analytes. The current dataset includes a greater number of plasma than CSF samples, highlighting an opportunity for future expansion. Additionally, while AMP PD captures detailed medication usage data, the potential confounding effects of medication on protein levels remain incompletely understood and warrant further investigation to support the development of clinically relevant proteomic biomarkers.

In conclusion, we have described the targeted and untargeted proteomics datasets generated in AMP® PD Release 4.0, including both raw and processed data. These resources offer powerful tools for investigating proteomic alterations in PD and may also yield insights into shared mechanisms across neurodegenerative disorders. By enabling researchers to integrate proteomics with multi‐omic and clinical data, AMP PD supports the discovery of novel diagnostic and therapeutic strategies aimed at improving outcomes for PD and related disorders.

## Author Roles

(1) Data Generation and Analysis: A. Conception, B. Organization, C. Execution; (2) Manuscript: A. Writing of the First Draft, B. Content Contribution C. Review and Critique; (3) A. Team Lead.

V.J.D.: 2A, 2B, 3A.

A.V.: 1A, 3A.

A.K. 1B, 1C.

N.S.: 1A, 1B, 1C, 2B.

R.P.: 1A, 1B.

P.B.: 1A, 1B, 1C, 2B.

J.E.V.: 3A.

M.Q.: 2B, 2C.

B.L.: 2C.

D.V.: 2C.

B.C.: 2D.

M.B.: 2C.

W.N.: 2C.

E.T.: 2B, 2C.

C.K.: 2B, 2C.

R.K.: 2B, 3A.

R.H.: 2C.

G.N.: 2C.

P.S.: 2C, 3A.

S.S.P.: 2C.

C.S.‐F.: 2B, 2C, 3A.

## Financial Disclosures of All Authors (for the Past 12 Months)

V.J.D.: No conflict of interest. Employed by Technome. Technome is contracted by the Foundation for National Institutes of Health (FNIH) for work on AMP® PD and AMP® PDRD. Technome is a sub‐awardee to a National Institutes of Health (NIH) Common Fund SysBio FAIRplex Award, awarded to Verily Life Sciences. A.V.: No conflict of interest. Employed by AbbVie Inc., owns AbbVie stocks. A.K.: Was an employee of AbbVie Inc. at the time of this study. He is a full‐time employee of Janssen R&D and may hold stock in Johnson & Johnson. N.S.: No conflict of interest. Employed by Cedars Sinai at the time of this study. R.P.: No conflict of interest. Employed by Cedars Sinai at the time of this study. Dr. P.B.: No conflict of interest. Employed by Tufts University, at the time of the study Dr P.B. was Employed by Sanofi. J.E.V.: No conflict of interest. Funding sources: Answer ALS from ALS Finding a Cure/Robert Packard Center for Finding a Cure and also 1U54CA260591–01 NIH; 2 U01 DK108314‐06 NIH; 1 R01 HL147351‐01A1 NIH; CTI: NCT04634539 NIH; 1 R01 HL155346‐01A1 NIH; 1R01AG089711–01 NIH; #HT94252510404 (DOD); 5U01DK124019–04 NIH, and U01NS115658 NIH. M.Q.: No conflict of interest. Employed by AbbVie Inc., owns AbbVie stocks. B.L. No conflict of interest. Employed by Technome. Technome is contracted by the FNIH for work on AMP® PD and AMP® PDRD. Technome is a sub‐awardee to an NIH Common Fund SysBio FAIRplex Award, awarded to Verily Life Sciences. D.V.: No conflict of interest. Employed by Technome. Technome is contracted by the FNIH for work on AMP® PD and AMP® PDRD. Technome is a sub‐awardee to a NIH Common Fund SysBio FAIRplex Award, awarded to Verily Life Sciences. B.C.: No conflict of interest. Employed by The Michael J. Fox Foundation for Parkinson's Disease. M.B.: No conflict of interest. Employed by Verily Life Sciences and owns Verily Life Sciences stock. W.N. No conflict of interest. Employed by Verily Life Sciences and owns Verily Life Sciences stock. E.T. No conflict of interest. Employed by Sanofi and owns Sanofi stock. C.K.: No conflict of interest. Employed by Sanofi and owns Sanofi stock. R.K.: No conflict of interest. Employed by Sanofi and owns Sanofi stock. R.H.: No conflict of interest. Employed by BMS and owns BMS stock and stock options. G.N.: No conflict of interest related to the research in this article. GSK is one of the private sponsors in the AMP‐PD Public–Private Consortia. GSK is one of the private sponsors in the AMP‐PD, AMP‐AD, and AMP‐ALS consortia. S.P.S.: No conflict of interest. Employed by Sanofi and owns Sanofi stock. S.R.P.: The author declares no conflicts of interest. The author is employed by the Foundation for the National Institutes of Health (FNIH), which convened and manages the public–private partnership that funded this research. The author also serves as the FNIH representative on the Neurosciences Forum at the National Academy of Sciences. In addition, the author is Principal Investigator on two Innovation Discretionary Awards from the Wellcome Trust (Grant Reference Numbers 220,664/A/20/Z and 220,664/Z/20/Z), which provide funding for the Accelerating Medicines Partnership in Schizophrenia, a public–private partnership convened and managed by the FNIH. C.S.‐F.: No conflict of interest. Employee of the National Institute of Neurological Disorders and Stroke.

## Supporting information


**Appendix S1.** Supplementary Information.

**Figure S1.** Venn diagrams illustrate the overlap between tissue types (cerebrospinal fluid and plasma) and assay platforms (Proteomics Data‐Independent Acquisition [PDIA] and Proteomics Proximity Extension Assay [PPEA]). Figure 1A depicts sample‐level overlap, with a total of 831 samples shared across tissue types and assay platforms. Figure 1B shows participant level overlap, with 237 participants contributing samples across all samples and platforms. To maximize the utility of the proteomics dataset, the study design intentionally incorporated overlap across both tissue types and assay platforms. As shown in Figure 1A,B, this design yielded substantial shared representation at both the sample and participant levels, facilitating integrative analyses across platforms and biospecimens.
**Figure S2.** Comparison of differentially expressed proteins between the Parkinson's Disease Biomarkers Project (PDBP) and Parkinson's Progression Markers Initiative (PPMI) cohorts in cerebrospinal fluid. Each cohort showed a relatively small number of significantly differentially expressed proteins, with only limited overlap between them.
**Figure S3.** Comparison of differentially expressed proteins between the Parkinson's Disease Biomarkers Project (PDBP) and Parkinson's Progression Markers Initiative (PPMI) cohorts in plasma reveals a limited number of significant proteins in each cohort with minimal overlap.
**Figure S4.** (A) Principal component analysis (PCA) of plasma proteomics data at month 0 (baseline) shows no distinct clustering between participants with or without a known Parkinson's disease (PD)‐associated mutation. The distribution along PC1 and PC2 indicates substantial overlap between the two groups, suggesting broadly similar proteomic profiles at this timepoint. (B) PCA of plasma proteomics data at month 24 shows no distinct clustering between participants with or without a known PD‐associated mutation. (C) PCA of plasma proteomics data at month 48 shows no distinct clustering regardless of PD mutation carrier status.
**Figure S5.** Plasma protein expression differences between participants with a known Parkinson's disease (PD)‐associated mutation and those without at three timepoints: month 0 (A), month 24 (not shown), and month 48 (B). Differential expression analysis identified significantly differentially expressed proteins at month 0 and month 48, while no significant differences were observed at month 24. This suggests temporal variation in proteomic signatures associated with mutation status.
**Figure S6.** Analysis of cerebrospinal fluid proteomics data comparing Parkinson's disease (PD) to other neurological disorders as classified by Accelerating Medicines Partnership® Parkinson's Disease (AMP® PD) clinical data. The volcano plot illustrates proteins that are significantly upregulated or downregulated in *Other neurological disorders* relative to PD.
**Figure S7.** This screenshot of the Terra platform ‘My Workspaces’ view displays the available Accelerating Medicines Partnership® Parkinson's Disease (AMP® PD) Community Provided Workspaces.

**Table S1.** Timepoint distribution of samples in Proteomics Data‐Independent Acquisition (PDIA) mass spectrometry‐derived data and PPEA proximity extension assay‐derived data.
**Table S2.** Mutations found in whole‐genome sequencing within Accelerating Medicines Partnership® Parkinson's Disease (AMP® PD) samples in Proteomics Data‐Independent Acquisition (PDIA) mass spectrometry‐derived data and PPEA proximity extension assay‐derived data.
**Table S3.** Breakdown of plasma untargeted proteomics sample counts for case, control, and other neurodegenerative disease diagnosis.
**Table S4.** Total number of plasma samples at months 0 (M0), 24 (M24), and 48 (M48), stratified by the presence or absence of a known Parkinson's disease‐associated mutation or variant *GBA1 (N370S [rs76763715]; T369M [rs75548401]; E326K [rs2230288]), LRRK2 (G2019S [rs34637584]; R1441C_T [rs33939927]; R1441G_G [rs33939927]), SNCA (A53T [rs104893877])* or *APOE ε4*.
**Table S5.** Getting Started Tier 2 – Proteomics Workspace notebooks within Accelerating Medicines Partnership® Parkinson's Disease (AMP® PD) Knowledge Portal in Terra, separated by coding language (Python or R).
**Table S6.** Proteomics Quality Control and Analysis Workspace notebooks within Accelerating Medicines Partnership® Parkinson's Disease (AMP® PD) Knowledge Portal in Terra.

## Data Availability

The data that support the findings of this study are openly available in the AMP® PD Knowledge Portal at https://amp-pd.org/.

## References

[1] Gobena S , Admassu B , Kinde MZ , Gessese AT . Proteomics and its current application in biomedical area: concise review. Sci World J 2024;2024:4454744. 10.1155/2024/4454744 PMC10894052 38404932

[2] Dayon L , Cominetti O , Affolter M . Proteomics of human biological fluids for biomarker discoveries: technical advances and recent applications. Expert Rev Proteomics 2022;19(2):131–151. 35466824 10.1080/14789450.2022.2070477

[3] Geyer PE , Hornburg D , Pernemalm M , et al. The circulating proteome horizontal line technological developments, current challenges, and future trends. J Proteome Res 2024;23(12):5279–5295. 39479990 10.1021/acs.jproteome.4c00586 PMC11629384

[4] Petrosius V , Schoof EM . Recent advances in the field of single‐cell proteomics. Transl Oncol 2023;27:101556. 36270102 10.1016/j.tranon.2022.101556 PMC9587008

[5] Bloem BR , Okun MS , Klein C . Parkinson's disease. Lancet 2021;397(10291):2284–2303. 33848468 10.1016/S0140-6736(21)00218-X

[6] Nalls MA , Blauwendraat C , Vallerga CL , et al. Identification of novel risk loci, causal insights, and heritable risk for Parkinson's disease: a meta‐analysis of genome‐wide association studies. Lancet Neurol 2019;18(12):1091–1102. 31701892 10.1016/S1474-4422(19)30320-5 PMC8422160

[7] Licker V , Turck N , Kovari E , et al. Proteomic analysis of human substantia nigra identifies novel candidates involved in Parkinson's disease pathogenesis. Proteomics 2014;14(6):784–794. 24449343 10.1002/pmic.201300342

[8] Leyns CEG , Prigent A , Beezhold B , et al. Glucocerebrosidase activity and lipid levels are related to protein pathologies in Parkinson's disease. NPJ Parkinsons Dis 2023;9(1):74. 37169750 10.1038/s41531-023-00517-w PMC10175254

[9] Steger M , Tonelli F , Ito G , et al. Phosphoproteomics reveals that Parkinson's disease kinase LRRK2 regulates a subset of Rab GTPases. elife 2016;5:5. 10.7554/eLife.12813 PMC4769169 26824392

[10] Petyuk VA , Yu L , Olson HM , et al. Proteomic profiling of the substantia nigra to identify determinants of Lewy body pathology and dopaminergic neuronal loss. J Proteome Res 2021;20(5):2266–2282. 33900085 10.1021/acs.jproteome.0c00747 PMC9190253

[11] Chelliah SS , Bhuvanendran S , Magalingam KB , Kamarudin MNA , Radhakrishnan AK . Identification of blood‐based biomarkers for diagnosis and prognosis of Parkinson's disease: a systematic review of proteomics studies. Ageing Res Rev 2022;73:101514. 34798300 10.1016/j.arr.2021.101514

[12] Hu L , Dong MX , Huang YL , et al. Integrated metabolomics and proteomics analysis reveals plasma lipid metabolic disturbance in patients with Parkinson's disease. Front Mol Neurosci 2020;13:80. 32714143 10.3389/fnmol.2020.00080 PMC7344253

[13] Karayel O , Virreira Winter S , Padmanabhan S , et al. Proteome profiling of cerebrospinal fluid reveals biomarker candidates for Parkinson's disease. Cell Rep Med 2022;3(6):100661. 35732154 10.1016/j.xcrm.2022.100661 PMC9245058

[14] Kaiser S , Zhang L , Mollenhauer B , et al. A proteogenomic view of Parkinson's disease causality and heterogeneity. NPJ Parkinsons Dis 2023;9(1):24. 36774388 10.1038/s41531-023-00461-9 PMC9922273

[15] Rutledge J , Lehallier B , Zarifkar P , et al. Comprehensive proteomics of CSF, plasma, and urine identify DDC and other biomarkers of early Parkinson's disease. Acta Neuropathol 2024;147(1):52. 38467937 10.1007/s00401-024-02706-0 PMC10927779

[16] Imam F , Saloner R , Vogel JW , et al. The Global Neurodegeneration Proteomics Consortium: biomarker and drug target discovery for common neurodegenerative diseases and aging. Nat Med 2025;31(8):2556–2566. 40665048 10.1038/s41591-025-03834-0 PMC12353841

[17] Sun BB , Chiou J , Traylor M , et al. Plasma proteomic associations with genetics and health in the UK Biobank. Nature 2023;622(7982):329–338. 37794186 10.1038/s41586-023-06592-6 PMC10567551

[18] Chen‐Plotkin AS , Albin R , Alcalay R , et al. Finding useful biomarkers for Parkinson's disease. Sci Transl Med 2018;10(454):eaam6003. 30111645 10.1126/scitranslmed.aam6003 PMC6097233

[19] Tsukita K , Sakamaki‐Tsukita H , Kaiser S , et al. High‐throughput CSF proteomics and machine learning to identify proteomic signatures for Parkinson disease development and progression. Neurology 2023;101(14):e1434–e1447. 37586882 10.1212/WNL.0000000000207725 PMC10573147

[20] Hepp DH , van Wageningen TA , Kuiper KL , et al. Inflammatory blood biomarkers are associated with long‐term clinical disease severity in Parkinson's disease. Int J Mol Sci 2023;24(19):14915. 37834363 10.3390/ijms241914915 PMC10573398

[21] Bartl M , Dakna M , Schade S , et al. Blood markers of inflammation, neurodegeneration, and cardiovascular risk in early Parkinson's disease. Mov Disord 2023;38(1):68–81. 36267007 10.1002/mds.29257

[22] Feng W , Beer JC , Hao Q , et al. NULISA: a proteomic liquid biopsy platform with attomolar sensitivity and high multiplexing. Nat Commun 2023;14(1):7238. 37945559 10.1038/s41467-023-42834-x PMC10636041

