# Supplementary material for: Accelerating Medicines Partnership® Parkinson's Disease Proteomics: A Comprehensive Resource for Advancing Parkinson's Disease Research
Source: Mov Disord. 2026 Feb 5;41(4):972–80. doi: 10.1002/mds.70183 (PMC13067336; doi:10.1002/mds.70183)
Supplement: Supplementary file 1 — Appendix S1. Supplementary Information. [file MDS-41-972-s001.docx]

**Appendix S1: Supplementary Information**

Targeted Proteomics Methods

Proteins were measured using Olink® Explore 1536 (Olink Proteomics AB, Uppsala, Sweden) according to the manufacturer's instructions. The technology behind the Olink protocol is based on Proximity Extension Assay (PEA) (Assarsson et al, 2014), coupled with readout via next-generation sequencing (NGS). The assay enables the detection of up to 1536 proteins in 90 samples simultaneously, using only 2.8 µL of serum/plasma sample. In brief, pairs of oligonucleotide-labeled antibody probes designed for each protein bind to their target, bringing the complementary oligonucleotides in close proximity and allowing for their hybridization. The addition of a DNA polymerase leads to the extension of the hybridized oligonucleotides, generating a unique protein identification “barcode”. Next, library preparation adds sample identification indexes and the required nucleotides for Illumina sequencing. Prior to sequencing using the Illumina® NovaSeq™ 6000/NextSeq™ 550/NextSeq™ 2000, libraries go through a bead-based purification step and the quality is assessed using the Agilent 2100 Bioanalyzer (Agilent Technologies, Palo Alto, CA). The raw output data is quality controlled, normalized and converted into Normalized Protein eXpression (NPX) values, Olink’s proprietary unit of relative abundance. Three internal controls are spiked into every sample and are used to monitor the performance of the three main steps in the protocol: the incubation control consists of a non-human antigen and its matching antibody probes, the extension control is an IgG antibody coupled to a pair of oligonucleotides that are always in proximity and the amplification control is a complete double strand DNA amplicon. In parallel with the samples, the protocol is performed on a set of external controls: Two sample controls of pooled plasma sample, three negative controls and three plate controls (PC). The plate controls are additional plasma samples that are used for normalization between plates. *NPX values are calculated in two steps: 1. ExtNPX(assay i, sample j) = log2(counts(assay i, sample j)/(counts(Extension control, sample j)) *(relate the counts to a known standard)* 2. NPX(assay i, sample j) = ExtNPX(assay i, sample j) – median (ExtNPX(PC, assay i)) *(plate standardization)*. **NPX values are calculated in three steps: 1. ExtNPX(assay i, sample j) = log2(counts(assay i, sample j)/(counts(Extension control, sample j)) *(relate the counts to a known standard)* 2. NPX(assay i, sample j) = ExtNPX(assay i, sample j) – median (ExtNPX(PC, assay i)) *(plate standardization)* 3. NPXintNorm(assay i, sample j) = (NPX(assay i, sample j) – plate median (NPX(assay i)) + global median (NPX(assay i)) *(corrects for nor-randomized samples)*. Limit of detection (LOD) is defined as 3 standard deviations (SD) above the median NPX of the negative controls. The SD is a predefined value. Detectability is reported as the percentage of samples above the LOD. The CV is calculated per assay under the assumption of a log-normal distribution. Quality control is performed per sample plate on both the samples (using the spiked internal controls) and the external controls. For all samples, the average counts may not fall below 500 counts and the incubation/amplification controls may not deviate from the median more than 0.3 NPX. Exceeding any of these criteria results in a QC warning for that sample. For the PCs and negative controls, the median of the triplicates may not exceed more than 5 SDs and 3 SDs, respectively, from predefined values for more than 10% of the assays. For the negative controls, only positive deviations are considered. Assays not fulfilling these criteria receive a QC warning. If the number of samples with a QC warning exceeds 1/6 of the samples on a plate and the median absolute deviation (MAD) for incubation or amplification control across all samples exceeds 0.3, the protocol is considered failed. All assay validation data (detection limits, intra- and inter-assay precision data, predefined values, etc.) are available on manufacturer's website ([www.olink.com](http://www.olink.com)).

*Use this for randomized studies.

** Use this for non-randomized studies.

Olink Bridging:

R Package *OlinkAnalyze* version 3.6.2 was used in this bridging analysis. The *OlinkAnalyze* collection of functions is developed by Olink and intended to facilitate analysis of proteomics data from Olink, primarily NPX data. Full details of the Olink Analyze R package can be found [on CRAN](https://cran.r-project.org/web/packages/OlinkAnalyze/index.html)**,** including information on code dependencies, limitations of the analysis, and other user guidance**.** The bridging and mean calculations were all conducted using R and were performed separately for cerebrospinal fluid data sets and the plasma data sets.

**Inclusion of Bridging Samples:**

For the purpose of applying the bridging protocol, these common bridging samples were identified using the dplyr R package to find the intersecting samples between Dataset D01 and Dataset D02. Upon manual review of results, a formatting difference was identified in 2 samples’ identifying information; once confirmed as a formatting error, this difference was manually corrected by the analyst to ensure the bridging samples were correctly processed by the bridging protocol. This list of bridging samples was used as an input to the olink_normalization.

**Bridging Data and QC**

As described in the *OlinkAnalyze* user manual, the bridging protocol applied involved the calculation of the median of the paired NPX differences per assay using the bridging samples. This determines the assay specific adjustment factor which is used to normalize NPX values between the two datasets. For Release D03, Dataset D01 was considered the reference dataset and therefore its NPX values remained unaltered. Dataset D02 was adjusted to the reference dataset based on the adjustment factors. The bridging samples themselves are adjusted to their average values and de-duplicated, resulting in one entry per bridging sample in the final bridged dataset. Therefore, users should expect that Dataset D03 contains *fewer* records than the combined unbridged results of the component projects.

In some cases, the application of the bridging protocol led to the duplication of the same sample x uniprot with two separate NPX values. In addition, there are cases in which there is a duplicate uniprot ID due to the same uniprot ID being in more than one Olink panel or there being two different Olink assays that map to that uniprot ID. In all of these cases, after the bridging protocol had been applied, the mean NPX value was calculated and used in the final data product, resulting in one NPX value for every uniprot ID for every sample ID.

## **AMP^®^ PD QC and Validation of Targeted Proteomics Data**

Three levels of QC metrics were applied to assess sample integrity without excluding any data points.

Outlier QC: NPX median and interquartile range were considered in generating the outlier QC. Samples were labeled as a “WARN” if both the median and IQR are outside of 3 standard deviations from the mean of the respective value.

Distribution QC: This column was calculated by taking into consideration the number of “WARN” values within a given sample. The Olink QC_Warning was given at the Uniprot level (the protein within each sample) and this considered the number of uniprot IDs labeled with a “WARN”.

Cumulative QC: Three QC columns were available to generate cumulative QC: Olink generated “QC_Warning”, and AMP PD generated “Distribution_QC” and “Outliers_QC.” The cumulative QC consisted of either a “PASS” or “FLAG” warning based on the three QC columns available. If all three QC columns contained “WARN”, the cumulative QC for this sample was a “FLAG.” The cumulative QC is available as an aggregate QC status, making it easier for users to identify flagged samples and investigate further. Note no samples were removed or excluded from the dataset, only flagged for user attention.

**Untargeted Proteomics Supplementary Methods**

Untargeted proteomics analysis was conducted on cerebrospinal fluid and blood plasma of both Parkinson's Disease patients and healthy participants in the PDBP and PPMI cohorts. 524 CSF samples and 522 plasma samples from the PDBP cohort mapping to 139 and 128 participants, respectively and 2283 CSF samples and 949 plasma samples from the PPMI cohort mapping to 481 and 179 participants, respectively were run through the assays. Analysis was conducted using DIA mass spectrometry-based proteomics utilizing trap-collision based disassociation to measure fragment intensity from processed peptide samples. The method requires fragment data to be rolled up to peptide data and then eventually protein intensity data that can be used in downstream analysis.

## ***Sample Preparation***

### **Cerebrospinal fluid (CSF)**

**CSF Protein Digestion -** Individual patient CSF samples and four CSF pooled samples from healthy individuals used as DCR were processed on a Beckman i7 automated liquid handling system in 96-well plate format using a method modified from our previous published methods for plasma and depleted plasma.^9-10^ Briefly, the CSF sample were denatured and reduced by incubating with agitation at 60°C for 60 minutes with 30 µL of 55% 2,2,2-trifluoro-ethanol (TFE, Sigma), 14mM Dithiothreitol (DTT, Sigma) and dissolve in 40mM NH_4_CO_3_ (Ammonium bicarbonate, Sigma). Samples were alkylated by adding 10µL of 50mM iodoacetamide (IAA, Sigma) and incubated in the dark at room temperature for 30 minutes. The alkylation was quenched by adding 10uL of 100mM DTT to samples followed by a 15-minute incubation with agitation at room temperature. Sample solutions were then diluted with 180µL of 100mM NH_4_CO_3_ to reduce TFE in solution concentration to ~6.5%. Samples were digested at 42°C for 4 hours in an Inheco incubator trypsin:protein ratio of 1:10. Sample digestion was quenched by adding 10 µL of 12.5% formic acid. The final peptide digest volume in the well was 265 µL, which was immediately sealed with foil adhesive seals and stored at -80°C until thawed for LC-MS analyses. Given the undigested CSF sample aliquot volumes of 25 µL and final peptide digest volumes of 265 µL as previously described, the following dilution scheme was used to target a peptide load of 500 ng for each sample injection.

**Adding iRT Peptide Standards to CSF peptides**- To prepare samples for LC-MS processing, sample peptides were thawed at room temperature and spun down, and 53µL were diluted into a second 96-well plate with 47µL 0.1% formic acid in water. 20µL of diluted peptides were then transferred to wells of a PCR plate containing 20µL of diluted commercial indexed retention time peptide standards (iRT) (Biognosys[WK1] ®). iRT standards were prepared by serial dilution, first diluting one vial of iRT peptides with 1000µL of 0.1% formic acid in water, then adding 90uL of the previous dilution to 910uL of 0.1% formic acid in water.

**CSF Sample Desalting** - CSF sample/iRT peptide mixtures were then desalted by loading 20µL of the mixture onto Evotips (EVOSEP, C18 disposable trap columns).

### **Plasma**

**Plasma Depletion** - Plasma samples were depleted using 96 well plate previously described by McArdle and Binek *et al.* (2022)*,* in which 14 most abundant proteins including albumin, immunoglobulins A, E, G, and M, kappa and lambda light chains, alpha-1-acidglycoprotein, alpha-1-antitrypsin, alpha-2- macroglobulin, apolipoprotein A1, fibrinogen, haptoglobin, and transferrin using the High Select Top 14 Abundant Protein Depletion Camel Antibody Resin (Thermo Fisher Scientific).^9^

The depletion protocol was previously described by McArdle and Binek et al., but here we scaled the method to allow for aliquoting depletion resin into 96-well plates in batches of 2 plates. Briefly, after equilibrating depletion resin at room temperature, the resin was poured into a 25mL pipetting reservoir affixed to an in-house made, 3D printed vortex attachment and shaken at 800 rpm to maintain a homogeneous resin slurry for consistent bead concentrations in each well of the 96-well plates containing diluted plasma samples (10 µL plasma diluted with 90 µL ammonium bicarbonate). Using a multichannel pipette, 300 µl of resin were added to all sample wells, and plates were sealed. Plates were then incubated in a plate shaker at 800 rpm for 1 hour at room temperature. Contents of the 96-well plates were then transferred to filter plates (Nunc) atop empty 96-well plates (Beckman Coulter), and the filter plate was sealed. The samples were then passed through the filter via centrifugation for 4 minutes at 100 rpm. Plates were then removed and rotated 180° then centrifuged for 4 minutes at 100 rpm. Resultant depleted proteins were then lyophilized via Speedvac and frozen at -80°C and stored until two more depleted plasma plates were ready for 4-plex tryptic digestion.

**Naïve Tryptic Digestion and Desalting** - Naïve plasma proteins were digested according to the protocol described by Fu *et al.* (2020)^2^. Naïve plasma tryptic peptide desalting was carried out using a positive pressure apparatus (Amplius Positive Pressure ALP, Beckman Coulter) mounted on the left side of the i7 workstation deck as described by Fu *et al.* (2020).^10^

**Naïve plasma iRT Peptide Addition** – Naïve plasma peptides were spiked 1:1 in MS vials with 1:20 diluted iRT peptides.

**Depleted Plasma Digestion, iRT Addition and Desalting –** Depleted plasma samples underwent the tryptic digestion procedure described by McArdle and Binek *et al.* (2022).^9^ Resultant peptides were further diluted (5 µL peptides with 120 µL 0.1% formic acid in H_2_O) then 25 uL of diluted peptides were spiked into 25uL of iRT standards (Biognosys^®^) that were diluted as described above for CSF Evotip loading. 20 uL of depleted plasma/iRT peptide mixtures were then desalted using Evotips.

## ***LC-MS Methods***

### **EVOSEP One LC System with Orbitrap Exploris 480 MS for CSF and Depleted Plasma**

Data independent acquisition (DIA-MS) was implemented by loading CSF sample peptides from Evotips onto an EVOSEP EV1106 Analytical Column (EVOSEP, C18 AQ, 1.9µm beads, 150µm ID, 15cm long with an EVOSEP One LC system). Samples were injected into an Orbitrap Exploris 480 mass spectrometer (ThermoScientific) with an average flow rate of 1.5 µl/min using the 30SPD EVOSEP method. The MS settings used for data acquisition are as follows: for Global MS Settings, expected LC peak width was set to 20 seconds and default charge state set at 2^+^. During the MS full scan data were collected in Profile form from 0 to 45 minutes over a scan range of 350-1400 m/z. Orbitrap resolution was set to 120k, while S-lens radio frequency was at 40%, normalized accumulated gain control (AGC) target was 300%, and maximum injection time was 45 milliseconds. DIA data were collected as Profile, scanning over a range of 200-1700 m/z with 50 isolation windows spanning 21 m/z with 1 m/z window overlap. Orbitrap resolution was 15k. Normalized collision energy was set to 28%, normalized AGC target was 100%, and maximum injection time was 22 milliseconds.

Depleted plasma tryptic peptides were separated by EVOSEP One LC System over a 45-minute gradient using the same column type as CSF and injected into an Orbitrap Exploris 480 MS using DIA-MS settings described by McArdle and Binek *et al.* (2022).^9^

### **Eksigent-415 LC System with Sciex Triple TOF 6600 MS for Naïve Plasma**

Naïve plasma peptides were analyzed using the method described by Holewinski *et al.* (2016).^11^ Briefly, samples data were acquired over a 60-minute gradient using a Eksigent-415 microflow LC system coupled to a Sciex Triple TOF 6600. DIA-MS methods used 100 variable windows over a chromatographic gradient of 60 minutes in the 400–1200 m/z range.

## ***Data Processing***

These were converted to profile mzML format using MSConvert for .raw conversion and Proteowizard for .wiff conversion.

Intensity data for peptide fragments was extracted from mzML files using the open source openSWATH workflow^1^ against the publicly available plasma library of the human twin population (February 2015) peptide assay library. Target and decoy peptides were then extracted, scored, and analyzed using the mProphet algorithm^2^ to determine scoring cut-offs consistent with a 1% false discovery rate (FDR). Peak group extraction data from each DIA file was combined using the “feature alignment” script, which performs data alignment and modeling analysis across an experimental data set^3^, and fragment-level data was normalized by MS2TIC.

Next, to obtain high-quality quantitative data, fragments from non-proteotypic peptides, i.e. peptides shared across different proteins^4^, were discarded. The large data set showed irregular patterns of missingness distribution, invoking the need for data filtering to discard fragments having ≥50% missingness across all three biological groups (done using metadata provided from the partial unblinding).

Technical bias from batching during digestion was corrected using comBat corrective technique. In the case of CSF samples, this was followed by Random Forest (RF) imputation. In the case of plasma, the depleted and native batch corrected data was combined at the fragment-level following the dual plasma workflow as specified in Zhang et al^7^.

The data was then processed using the mapDIA software to roll up fragment-level data to peptide and protein levels. PPMI CSF batch corrected and imputed files are provided. PPMI Plasma batch corrected files are provided.

Group Authorships

AMP PD Proteomics Working Group

- Samantha Hutton (MJFF)
- Amilcar Flores-Morales (Sanofi US Services Inc)
- Andy Christoforou (BMS)
- Bailin Zhang (Sanofi US Services Inc)
- Barry Landin (Technome)
- Bradford Casey (MJFF)
- Christine Swanson-Fischer (NINDS)
- Stacey Adam (FNIH)
- Howard Schulman (KOL)
- Lin An (Sanofi US Services Inc)
- Lyn Jakeman (NINDS)
- Marcus Bantscheff (GSK)
- Mark Frasier (MJFF)
- Pablo Sardi (Sanofi US Services Inc)
- Aparna Vasanthakumar (AbbVie)
