# Supplementary material for: Accelerating Medicines Partnership® Parkinson's Disease Proteomics: A Comprehensive Resource for Advancing Parkinson's Disease Research
Source: Mov Disord. 2026 Feb 5;41(4):972–80. doi: 10.1002/mds.70183 (PMC13067336; doi:10.1002/mds.70183)
Supplement: Supplementary file 2 — Figure S1. Venn diagrams illustrate the overlap between tissue types (cerebrospinal fluid and plasma) and assay platforms (Proteomics Data‐Independent Acquisition [PDIA] and Proteomics Proximity Extension Assay [PPEA]). Figure 1A depicts sample‐level overlap, with a total of 831 samples shared across tissue types and assay platforms. Figure 1B shows participant level overlap, with 237 participants contributing samples across all samples and platforms. To maximize the utility of the proteomics dataset, the study design intentionally incorporated overlap across both tissue types and assay platforms. As shown in Figure 1A,B, this design yielded substantial shared representation at both the sample and participant levels, facilitating integrative analyses across platforms and biospecimens. Figure S2. Comparison of differentially expressed proteins between the Parkinson's Disease Biomarkers Project (PDBP) and Parkinson's Progression Markers Initiative (PPMI) cohorts in cerebrospinal fluid. Each cohort showed a relatively small number of significantly differentially expressed proteins, with only limited overlap between them. Figure S3. Comparison of differentially expressed proteins between the Parkinson's Disease Biomarkers Project (PDBP) and Parkinson's Progression Markers Initiative (PPMI) cohorts in plasma reveals a limited number of significant proteins in each cohort with minimal overlap. Figure S4. (A) Principal component analysis (PCA) of plasma proteomics data at month 0 (baseline) shows no distinct clustering between participants with or without a known Parkinson's disease (PD)‐associated mutation. The distribution along PC1 and PC2 indicates substantial overlap between the two groups, suggesting broadly similar proteomic profiles at this timepoint. (B) PCA of plasma proteomics data at month 24 shows no distinct clustering between participants with or without a known PD‐associated mutation. (C) PCA of plasma proteomics data at month 48 shows no distinct clusterin [file MDS-41-972-s003.docx]

Supplementary Figures


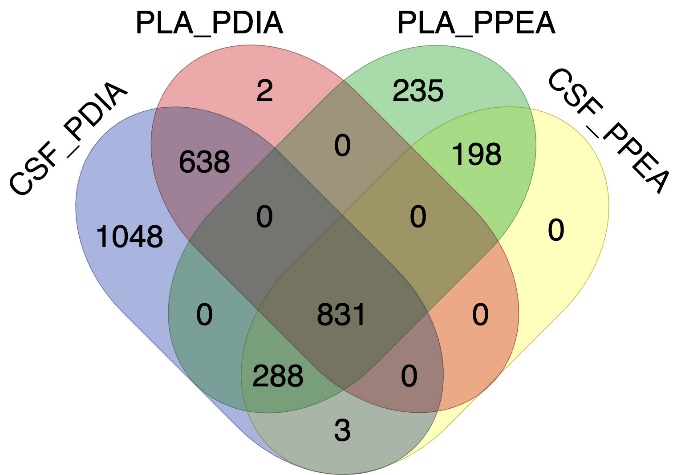

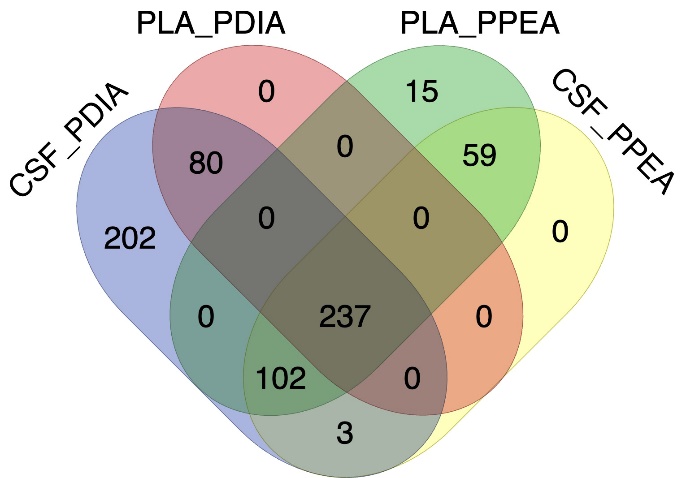


Supplementary Figure 1. Venn diagrams illustrate the overlap between tissue types (CSF and plasma) and assay platforms (PDIA and PPEA). Figure 1a depicts sample-level overlap, with a total of 831 samples shared across tissue types and assay platforms. Figure 1b shows participant level overlap, with 237 participants contributing samples across all samples and platforms. To maximize the utility of the proteomics dataset, the study design intentionally incorporated overlap across both tissue types and assay platforms. As shown in Figures 1a and 1b, this design yielded substantial shared representation at both the sample and participant levels, facilitating integrative analyses across platforms and biospecimens.


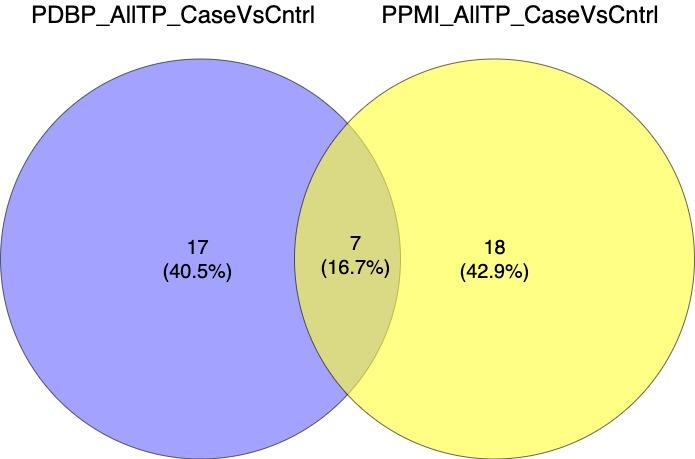


Supplementary Figure 2. Comparison of differentially expressed proteins between the PDBP and PPMI cohorts in CSF. Each cohort showed a relatively small number of significantly differentially expressed proteins, with only limited overlap between them.


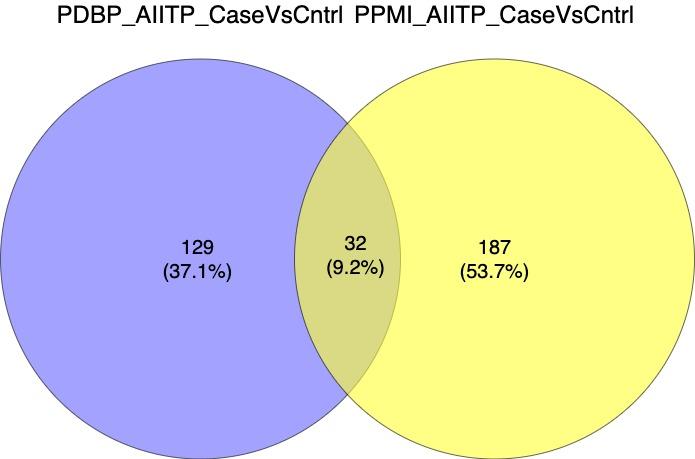


Supplementary Figure 3. Comparison of differentially expressed proteins between the PDBP and PPMI cohorts in plasma reveals a limited number of significant proteins in each cohort with minimal overlap.


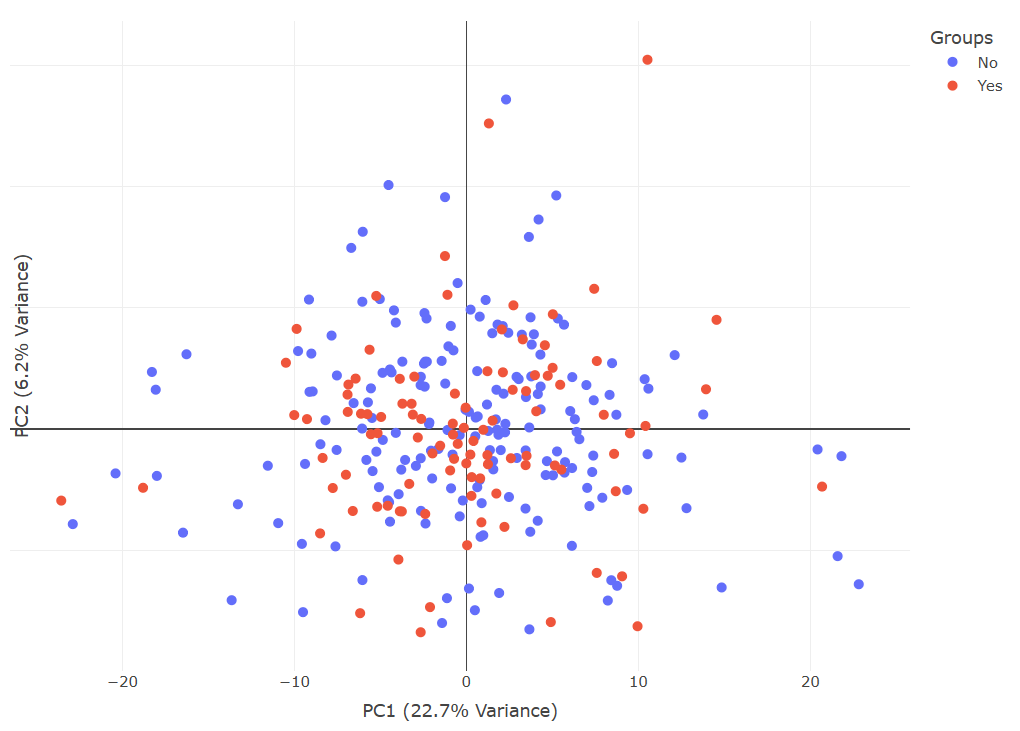


Supplementary Figure 4a. Principal Component Analysis (PCA) of plasma proteomics data at month 0 (baseline) shows no distinct clustering between participants with or without a known PD–associated mutation. The distribution along PC1 and PC2 indicates substantial overlap between the two groups, suggesting broadly similar proteomic profiles at this timepoint.


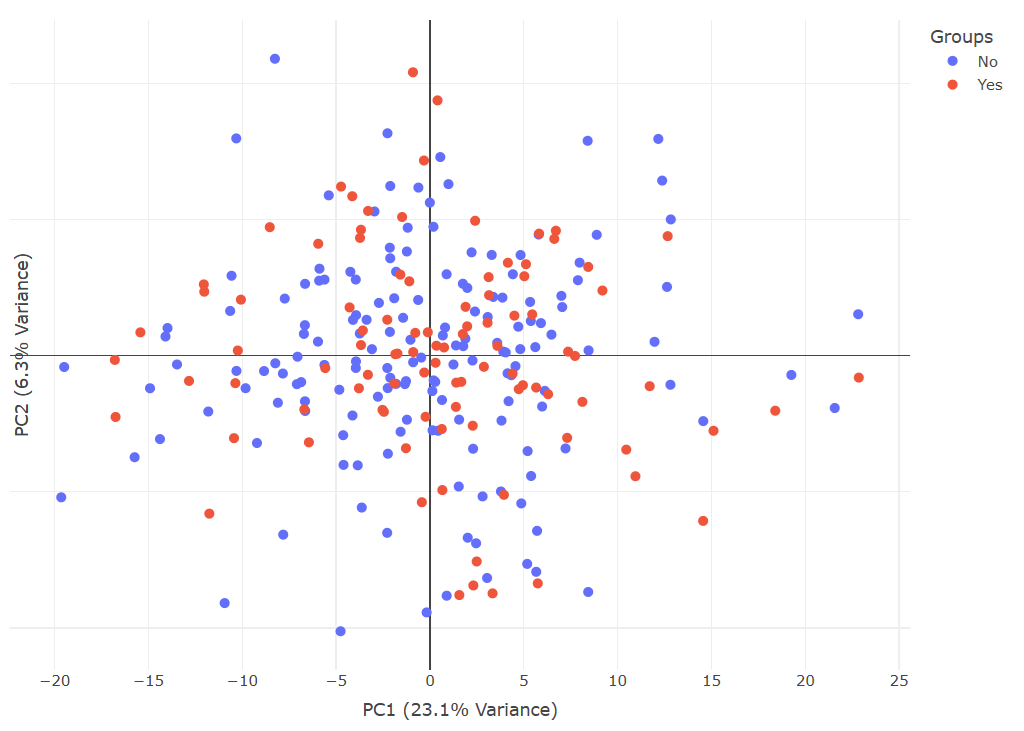


Supplementary Figure 4b: PCA of plasma proteomics data at month 24 shows no distinct clustering between participants with or without a known PD–associated mutation.


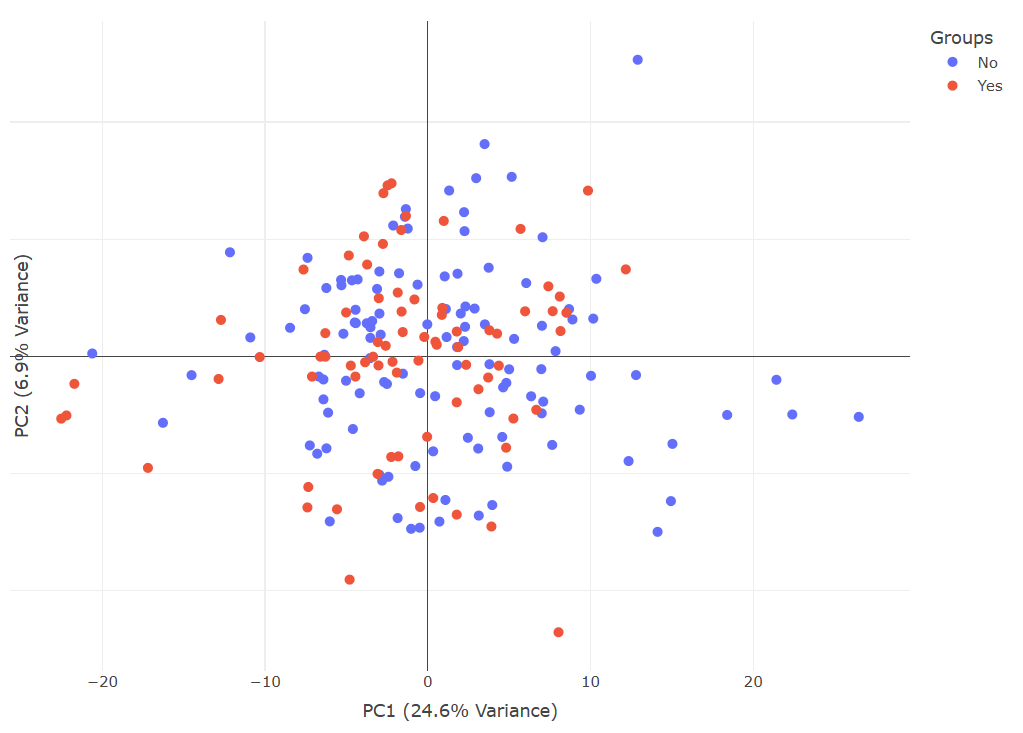


Supplementary Figure 4c: PCA of plasma proteomics data at month 48 shows no distinct clustering regardless of PD mutation carrier status.


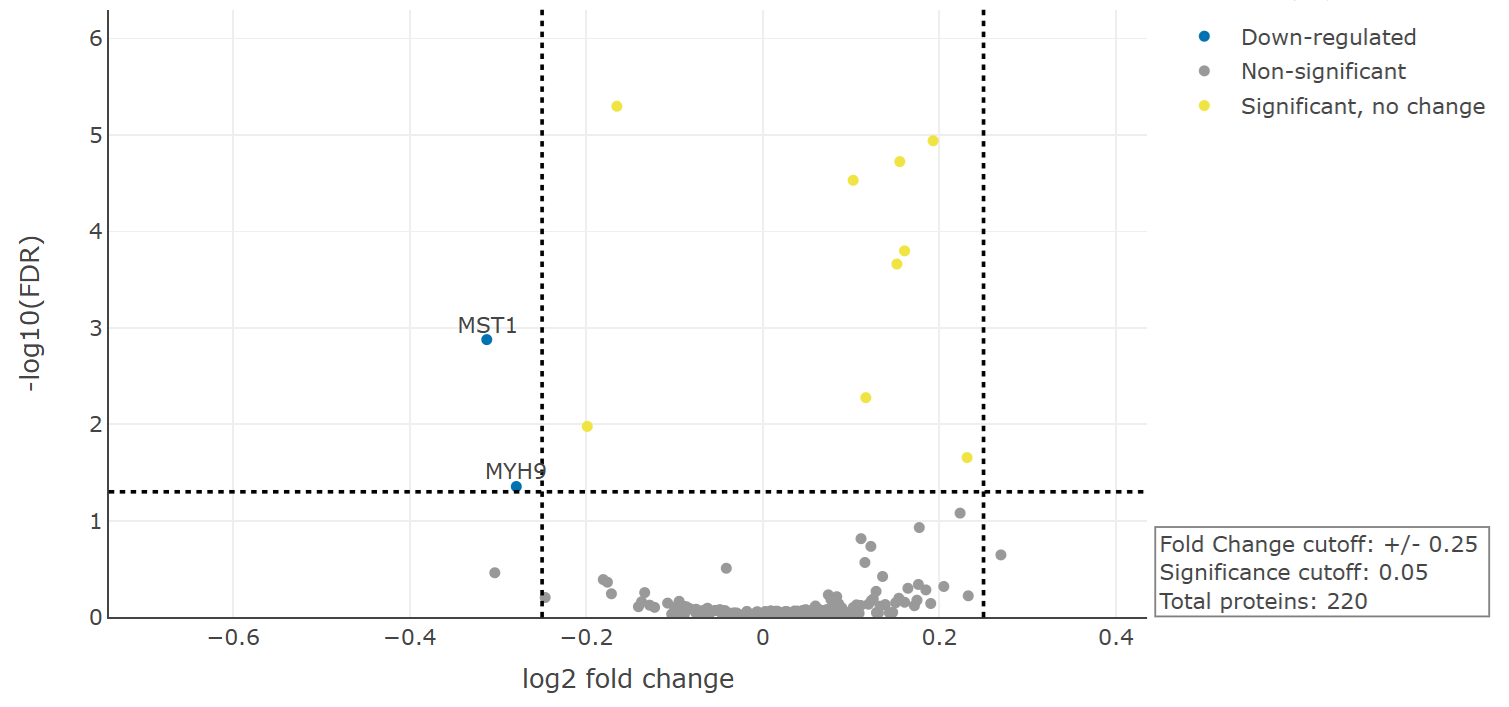


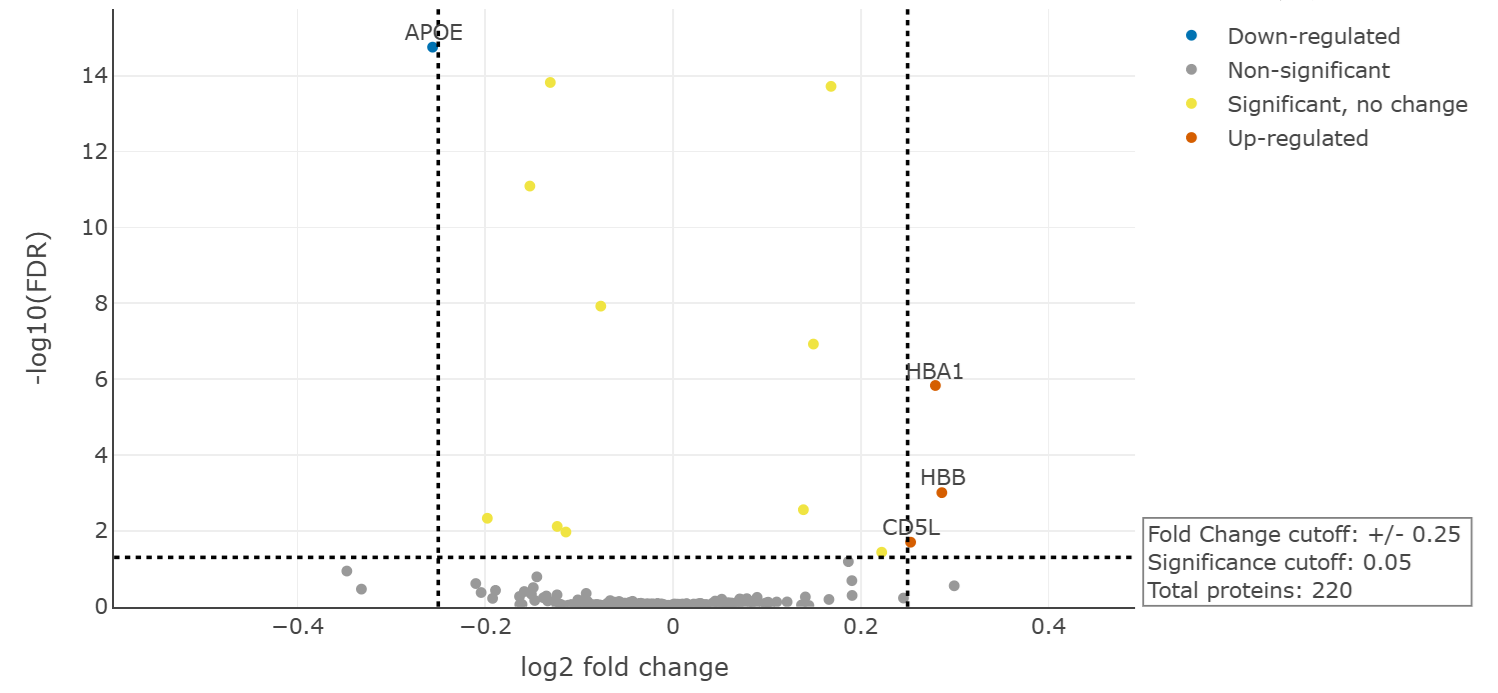
Supplementary Figure 5. Plasma protein expression differences between participants with a known PD-associated mutation and those without at three timepoints: month 0 (a), month 24 (not shown), and month 48 (b). Differential expression analysis identified significantly differentially expressed proteins at month 0 and month 48, while no significant differences were observed at month 24. This suggests temporal variation in proteomic signatures associated with mutation status.


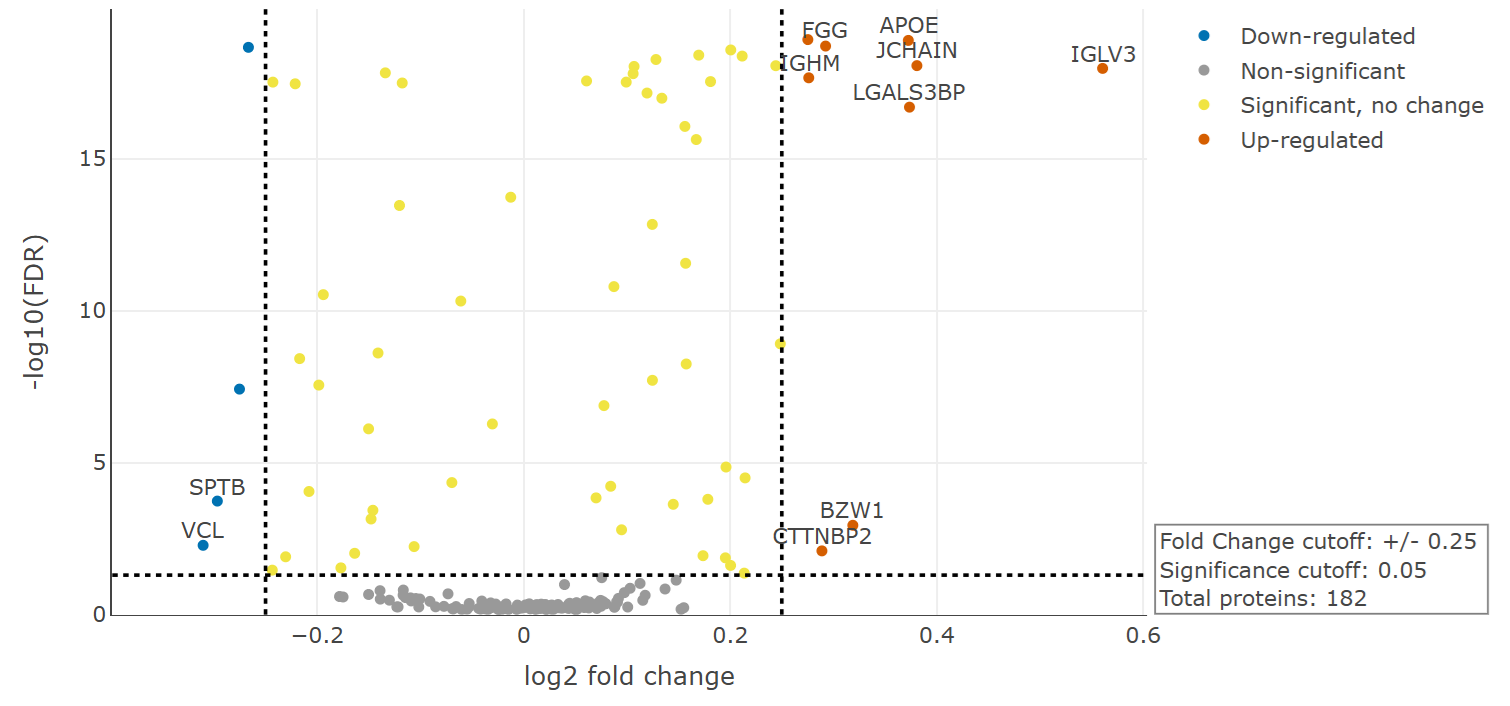
Supplementary Figure 6. Analysis of CSF proteomics data comparing PD to other neurological disorders as classified by AMP^®^ PD clinical data. The volcano plot illustrates proteins that are significantly upregulated or downregulated in *Other neurological disorders* relative to *PD*.


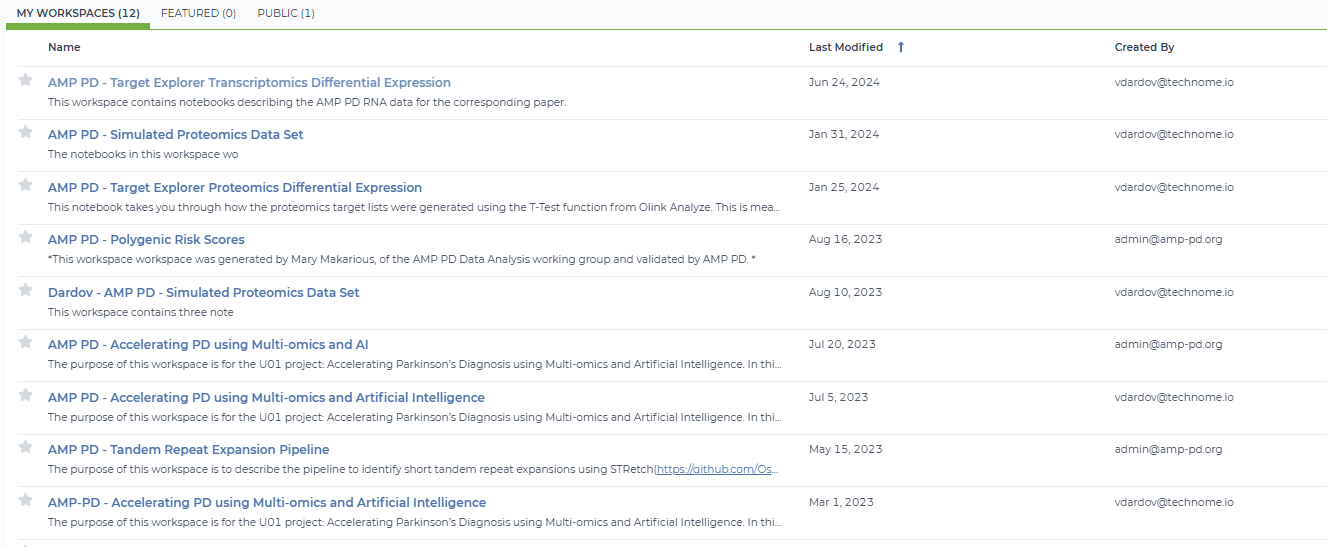


Supplementary Figure 7. This screenshot of the Terra platform “My Workspaces” view displays the available AMP^®^ PD Community Provided Workspaces.
