# Supplementary material for: Accelerating Medicines Partnership® Parkinson's Disease Proteomics: A Comprehensive Resource for Advancing Parkinson's Disease Research
Source: Mov Disord. 2026 Feb 5;41(4):972–80. doi: 10.1002/mds.70183 (PMC13067336; doi:10.1002/mds.70183)
Supplement: Supplementary file 3 — Table S1. Timepoint distribution of samples in Proteomics Data‐Independent Acquisition (PDIA) mass spectrometry‐derived data and PPEA proximity extension assay‐derived data. Table S2. Mutations found in whole‐genome sequencing within Accelerating Medicines Partnership® Parkinson's Disease (AMP® PD) samples in Proteomics Data‐Independent Acquisition (PDIA) mass spectrometry‐derived data and PPEA proximity extension assay‐derived data. Table S3. Breakdown of plasma untargeted proteomics sample counts for case, control, and other neurodegenerative disease diagnosis. Table S4. Total number of plasma samples at months 0 (M0), 24 (M24), and 48 (M48), stratified by the presence or absence of a known Parkinson's disease‐associated mutation or variant GBA1 (N370S [rs76763715]; T369M [rs75548401]; E326K [rs2230288]), LRRK2 (G2019S [rs34637584]; R1441C_T [rs33939927]; R1441G_G [rs33939927]), SNCA (A53T [rs104893877]) or APOE ε4. Table S5. Getting Started Tier 2 – Proteomics Workspace notebooks within Accelerating Medicines Partnership® Parkinson's Disease (AMP® PD) Knowledge Portal in Terra, separated by coding language (Python or R). Table S6. Proteomics Quality Control and Analysis Workspace notebooks within Accelerating Medicines Partnership® Parkinson's Disease (AMP® PD) Knowledge Portal in Terra. [file MDS-41-972-s002.docx]

Supplementary Tables


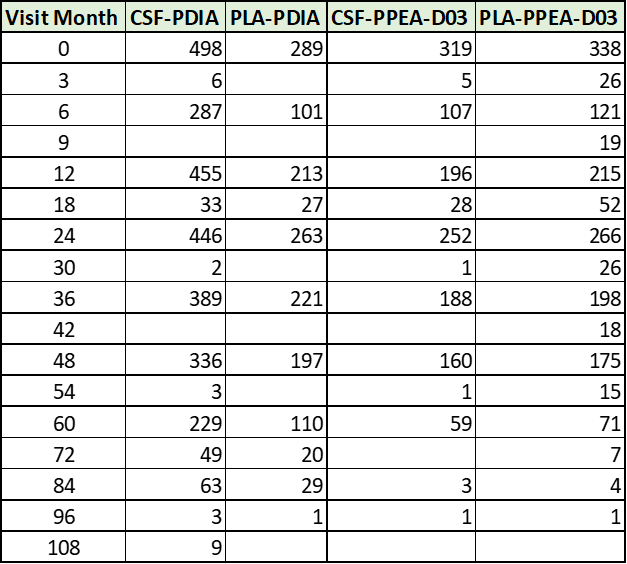


Supplementary Table 1. Timepoint distribution of samples in PDIA mass spectrometry derived data and PPEA proximity extension assay derived data.


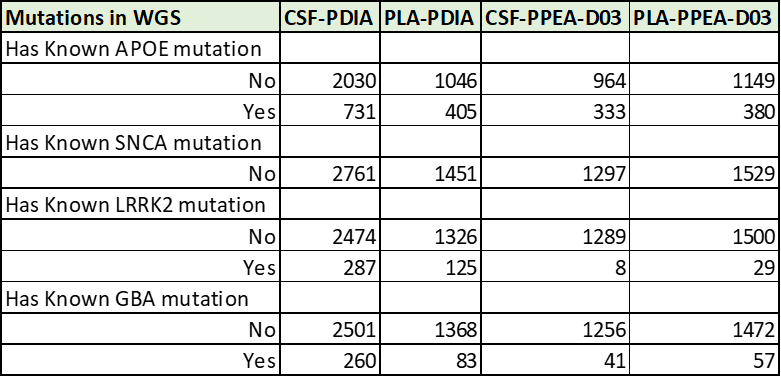


Supplementary Table 2. Mutations found in WGS within AMP^®^ PD samples in PDIA mass spectrometry derived data and PPEA proximity extension assay derived data.


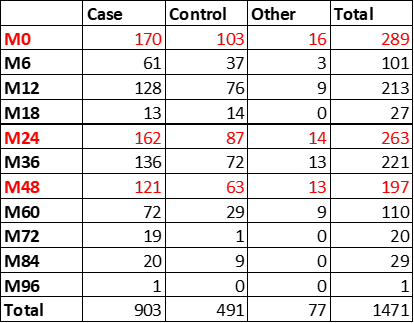


Supplementary Table 3. Breakdown of plasma untargeted proteomics sample counts for case, control and other neurodegenerative disease diagnosis.


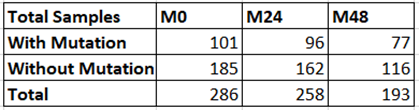


Supplementary Table 4. Total number of plasma samples at months 0 (M0), 24 (M24), and 48 (M48), stratified by the presence or absence of a known Parkinson’s disease-associated mutation or variant (*GBA1 ((N370S (rs76763715); T369M (rs75548401); E326K (rs2230288)), LRRK2 ((G2019S (rs34637584); R1441C_T (rs33939927); R1441G_G (rs33939927)), SNCA (A53T (rs104893877)*, or *APOE ε4*).

| **Python** | **R** | **R Studio** |
| --- | --- | --- |
| - Py3 - Proteomics - 00 - Untargeted Proteomics Directory | - R - Proteomics - 00 - Untargeted Proteomics Directory | - R - Proteomics - R Studio Start Here |
| - Py3 - Proteomics - 01 - Start Here Untargeted | - R - Proteomics - 01 - Start Here |  |
| - Py3 - Proteomics - 01 - Start Here | - R - Proteomics - 01 - Start Here Untargeted |  |
| - Py3 - Proteomics - 02 - Load Proteomics Data from BigQuery | - R - Proteomics - 02 - Load Proteomics Data from BigQuery |  |
| - Py3 - Proteomics - 03 - Merge with Demographics and Visualizations | - R - Proteomics - 03 - Merge with Demographics and Visualizations |  |
| - Py3 - Proteomics - 04 - Untargeted Data Assign Case Control and Downloading | - R - Proteomics - 04 - Targeted Data Filtering Assign Case Control and Downloading |  |
|  | - R - Proteomics - 04 - Untargeted Data Filtering Assign Case Control and Downloading |  |
|  | - R - Proteomics - Merge Proteomics with Global Sample Inventory |  |

Supplementary Table 5. Getting Started Tier 2 – Proteomics Workspace notebooks within AMP PD Knowledge Portal in Terra, separated by coding language (Python or R).

| **QC** | **Analysis** |
| --- | --- |
| - R- Proteomics- QCPlots | - R- Proteomics – Targeted Proteomics PCA |
|  | - R- Proteomics – Untargeted Proteomics PCA |
|  | - R- Proteomics – Box Plots of Selected Proteins |
|  | - R- Proteomics – APOE case study |

Supplementary Table 6. Proteomics QC and Analysis Workspace notebooks within AMP PD Knowledge Portal in Terra.
